# Polyphenols—Ensured Accessibility from Food to the Human Metabolism by Chemical and Biotechnological Treatments

**DOI:** 10.3390/antiox12040865

**Published:** 2023-04-03

**Authors:** Oana Lelia Pop, Ramona Suharoschi, Sonia Ancuța Socaci, Elaine Berger Ceresino, Achim Weber, Carmen Gruber-Traub, Dan Cristian Vodnar, Anca Corina Fărcaș, Eva Johansson

**Affiliations:** 1 Department of Food Science, University of Agricultural Sciences and Veterinary Medicine, 400372 Cluj-Napoca, Romania; 2 Molecular Nutrition and Proteomics Laboratory, Institute of Life Sciences, University of Agricultural Sciences and Veterinary Medicine, 400372 Cluj-Napoca, Romania; 3 Department of Plant Breeding, The Swedish University of Agricultural Sciences, P.O. Box 190, SE-234 22 Lomma, Sweden; 4 Innovation Field Functional Surfaces and Materials, Fraunhofer Institute for Interfacial Engineering and Biotechnology, Nobelstraße 12, 70569 Stuttgart, Germany

**Keywords:** polyphenols, bioavailability, bio-accessibility, bioactivity, antioxidant, biotechnological, chemical treatment

## Abstract

Polyphenols are plant-based compounds famous for their positive impact on both human health and the quality of food products. The benefits of polyphenols are related to reducing cardiovascular diseases, cholesterol management, cancers, and neurological disorders in humans and increasing the shelf life, management of oxidation, and anti-microbial activity in food products. The bioavailability and bio-accessibility of polyphenols are of the highest importance to secure their impact on human and food health. This paper summarizes the current state-of-the-art approaches on how polyphenols can be made more accessible in food products to contribute to human health. For example, by using food processing methods including various technologies, such as chemical and biotechnological treatments. Food matrix design and simulation procedures, in combination with encapsulation of fractionated polyphenols utilizing enzymatic and fermentation methodology, may be the future technologies to tailor specific food products with the ability to ensure polyphenol release and availability in the most suitable parts of the human body (bowl, intestine, etc.). The development of such new procedures for utilizing polyphenols, combining novel methodologies with traditional food processing technologies, has the potential to contribute enormous benefits to the food industry and health sector, not only reducing food waste and food-borne illnesses but also to sustain human health.

## 1. Introduction

High-quality food with a functional, curative, and beneficial effect on human metabolism is increasingly desired worldwide. In this context, polyphenols which are present in a variety of food, e.g., in fruits and vegetables, tea, coffee, chocolate, whole grains, red wine, food by-products, and wastes [1,2,3,4], have received increasing attention for their positive impact on human health due to their bioactive properties, mainly antioxidant and anti-inflammatory ones [5,6,7,8,9,10]. Plenty of investigations have reported a positive effect from the consumption of polyphenol-rich food on incidences of chronic illnesses such as cardiovascular disease [11], diabetes mellitus [12], and cancer [13]. Furthermore, the anti-microbial and antifungal properties that polyphenols contribute to food items are worthy of being mentioned [9]. Plant-based polyphenols show a high variety of structures and can be classified using a number of schemes into a variety of groups; although the common feature of all phenolic compounds is that they possess an aromatic ring with at least one hydroxyl substituent [6]. The bioavailability (bio-accessibility, absorption, and transportation) of polyphenols for humans is affected by their chemical structures, but also by food matrices, their binding to other molecules, processing techniques used, and the host’s metabolism [14]. An increased understanding of how specific food biotechnologies and processing techniques (thermal treatment, chemical binding, enzymatic treatment, fermentation, and encapsulation) influence the bio-accessibility, bioavailability, and properties of the polyphenols will contribute to novel opportunities and perspectives of their uses. These understandings will lead to new functional foods, ingredients, or additives simultaneously as the increasing demand for health-promoting food is met. Here, we are reviewing the current state-of-the-art and future opportunities for the impact of polyphenols on food characteristics and human health. Furthermore, we elucidate how polyphenols can be utilized in the food industry and for human nutrition by understanding and applying specific and targeted processes.

## 2. Food Biotechnology and Conventional Food Processing for Human Well-Being

Food production and processing is a major business sector which contributes substantially to the world economy and includes various aspects such as the cultivation of raw material, food processing, distribution, marketing and selling, and food impact on human well-being and health [15]. The world population is currently approaching 7.9 billion, which is an increase of 1 billion during the last 10 years [16], and is predicted to reach 9.9 billion in 2050, which increases the demand on the food system to feed us all. Novel technologies in the food system, including the food biotechnology area, sustain and facilitate food production and contribute to fulfilling consumer demand on the quantity and quality of food. Food biotechnology encompasses a large array of technologies including the use of (i) genetically modified/engineered organisms (GMO), (ii) micro-organisms for the production of single-cell proteins, (iii) treatment of food sources or matrices with enzymatic, fermentation, and/or encapsulation techniques, (iv) evaluation of plant or food material through -omics (e.g., genomics, proteomics) technologies, (v) biosensors to evaluate quality aspects of the food, (vi) nanotechnology, etc. [17,18,19]. The emerging and novel food biotechnological methods are used along the whole food chain, i.e., from farm to fork. A range of benefits have been reported from the use of these novel food biotechnological methods, including a decrease in the use of pesticides and herbicides, an increase in production yield, an increase in functional foods on the market, the recovery of valuable compounds from food wastes, safer foods, a reduced production cost, and more salable food [20]. However, an increase in consumer awareness and criticism against non-natural and GMO production of food is also reported [21,22]. Here we are focusing on how novel food biotechnological methods can contribute to the human health perspective on the availability of polyphenols from food consumption.

## 3. Polyphenols in the Food Industry and Human Health

As time passes, consumers become more conscious of their nutrition and eating habits, and with these changes, the demand for products that satisfy the new concerns grows. One of the most discussed concerns is for preserving the bioactive compounds of fruits and vegetables and maximizing their potential [23]. Polyphenols’ main property is related to the fact that they can prevent the oxidative stress affecting and altering the cells [5,24]. Waste and by-products obtained from fruits and vegetables present significant potential sources for polyphenol content, and fermentation is a technology that can help capitalize on them [24,25,26].

**Figure 1:**
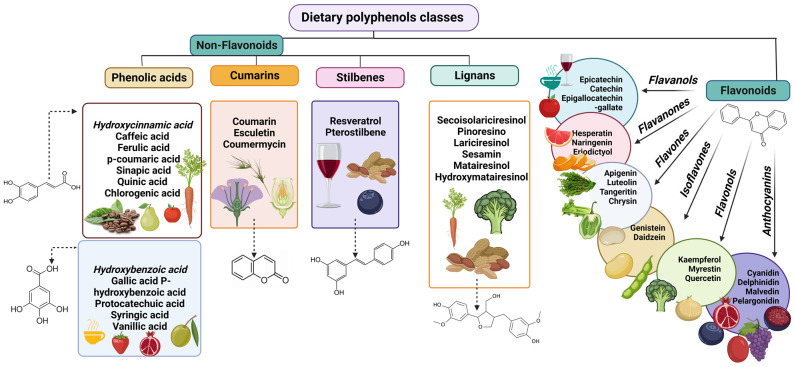
Polyphenol classification.

Polyphenols are valuable molecules, both for plant and human health and for the food industry, and they can be divided into two major classes–the non-flavonoid and the flavonoids polyphenols (Figure 1) [6]. The classification of polyphenols into different types is mainly based on their chemical structures.

In plants, polyphenols play an outstanding role in adaptation processes, e.g., they protect or heal in processes such as wounding, infection, and exposure to UV radiation [6]. Fruits, berries, and vegetables are rich sources of phenolic compounds in the diet [27]. Their polyphenols are mostly soluble, unbound, and highly bioaccessible, with only approximately one-quarter of the total amount attached to the plant and food matrix [28]. Fruits are particularly rich in hydroxycinnamic acids, which are rarely found in their free form, but instead as glycosylated derivatives or esters of quinic acid, shikimic acid, and tartaric acid [4]. In grains such as rice, wheat, oat, and corn, the phenolic acid content is also generally high, with ferulic acid (FA) being the most abundant among them [29]. However, the phenolic compounds in grains are bound to a much higher degree; 62% to 75% is commonly reported [30]. In particular, the bran of cereals is rich in FA, although esterified to the hemicellulose [4].

When consumed, some of the polyphenols, e.g., anthocyanin, are absorbed in the small intestine in the human body. Other polyphenols, especially in the glycoside form, need to be hydrolyzed by digestive enzymes, before they can be absorbed by epithelial cells [31,32]. For the food industry, polyphenols are important actors, positively prolonging the shelf life of food products (through their antioxidant, antibacterial, and antifungal activities) [5,9]. Simultaneously, polyphenols contribute with a positive impact on human health (through prebiotic, anti-platelet, anti-inflammatory and anti-proliferative actions) [7,31]. Polyphenols of plant origin are also experienced by consumers as natural ingredients, and the food industry can use them to replace chemical additives, thereby increasing the consumer value of food products [33]. Thus, different polyphenols have been applied on food products through spraying, dipping, coating, and have even been used on the surface of fresh food as functional packages. By such treatments, polyphenols have shown their capacity as protective agents by (i) delaying the action of free radicals, thereby prolonging the shelf life and security of the product [34,35] and (ii) by contributing anti-microbial and antifungal properties [36] through an impact on the permeability and integrity of the cell membranes, leading to growth inhibition of food-spoilage microorganisms (i.e., *Salmonella* spp., *Listeria monocytogenes*, *Escherichia coli*, *Aspergillus*, *Fusarium*, etc.).

Despite the many studies on the effects of polyphenols on the human body, the positive effects reported are not completely understood. In principal, a positive correlation has been proven between intake of polyphenols through food and human cardiovascular health [11]. Recently, a ten year follow-up study proved clearly that a high intake of flavonoids (found in cherries, chocolate, olives, coffee, and apples) reduces the incidence of cardiovascular diseases by almost 50% [37]. Furthermore, a positive correlation has been noted between tannic acid consumption and good endothelial function, implicitly regulating blood pressure and hemostasis of the human body, reducing inflammation, and increasing HDL cholesterol level [38]. Additionally, polyphenol consumption was shown to modulate the immune response through cytokine production and inhibition of pro-inflammatory gene expression, effects that were positive for human health. Intake of polyphenols (resveratrol) through red wine consumption in adequate amounts was correlated to a significant protective effect on the cardiovascular system through the anti-inflammatory activity of these phenols [39]. Likewise, a reduction of the expression of the inflammatory cytokines was observed with the consumption of curcumin, a non-flavonoid polyphenol found in turmeric, mustard, and curry powders [40]. A study conducted in 2018 showed how macrophages, which are the direct initiators of an inflammatory response, are suppressed by polyphenols such as the punicalagin from pomegranates [41]. A study on quercetin has both proven the broad impact of this phenolic compound on the human metabolism through its antidiabetic, anti-inflammatory, antioxidant, anti-microbial, anti-Alzheimer’s, antiarthritic, as well as cardiovascular and wound-healing effects, but also that nanoparticles of the phenolic compound contributed to better human health due to an increased bioavailability [42]. In fact, the suggested antioxidative action of the polyphenols may be the explanation of their anti-inflammatory effects in the human body, as protein oxidation is known to induce inflammation [43].

As discussed, polyphenols have potent anti-inflammatory and immunomodulatory properties, which largely account for their anticancer benefits. The NF-κB family of transcription factors controls the growth and maintenance of immune system cells and tissues as well as innate and adaptive immune responses. NF-κB dysregulation can cause cancer, chronic inflammation, and autoimmune disorders. By inhibiting NF-κB, polyphenol compounds can have anti-oxidant and immunomodulatory actions, which prevent tumor incidence and growth [44]. Cancer is one of the most dangerous illnesses to people’s health. In the fight to prevent and to combat this devastating illness it is vital to produce low-toxic anti-cancer chemicals due to the adverse effects brought on by chemotherapy and radiotherapy [45,46]. By encouraging apoptosis, controlling autophagy, obstructing proliferation and migration, and other mechanisms, polyphenols can be effective in the treatment of cancer. The mechanisms also involve epigenetic regulation. For instance, the plant polyphenol curcumin modulated tumor-related miRNA, produced global hypomethylation, and reduced the production of histone deacetylases (HDACs), which prevented cancer cells from proliferating, metastasizing, and forming new blood vessels while also inducing apoptosis [47,48,49,50]. Few in vivo studies have been done, and most research on the anticancer properties of various polyphenols has been done on preclinical animal models and in vitro cancer cells.

A well-defined principle, in a circular economy, is the valorization of food wastes and by-products is a desiderate. Plant-based food wastes and by-products (leaves, peels, pomace, seeds, and shells) can be an important source of polyphenols [34,51,52] and thus can help reduce inflammation and sustain the fight against oxidative damage [53].

The positive impact of polyphenols on food properties, combined with their human immunomodulatory role, as sustained by various in vitro and in vivo studies, calls for a sustainable and efficient increase in the use of these valuable plant-based compounds by the food industry. To prevent food spoilage and human diseases, polyphenols should be utilized in different and tailored compound mixtures (different polyphenols have various and multiple actions, and they might also be bound to other metabolites). Additionally, actions of polyphenols are not just dose-dependent, but their bioavailability and accessibility is a key of their impact. Overall, processes that ensure the maximum potential of the polyphenols for bio-accessibility and bioavailability in different matrixes and systems are worth being investigated and applied.

## 4. Techniques That Boost Polyphenols Availability

For human consumption, an increase in the bio-accessibility of phytochemicals bound to the food matrix is highly important. Phytochemicals are either covalently or non-covalently bound to the food matrix, and the total amount of phytochemicals in the food is of no relevance if they are not bioavailable, i.e., released from the food matrix for uptake after digestion in the intestine at consumption. Studies have indicated that a decrease in bound phenolics in the food results in a proportional increase in antioxidant effects from the food intake [30]. Polyphenols are usually located in the pectocellulosic wall of plant cells [54]. Thus, various measures impacting on the cell wall, also affect availability of the phenolic compounds. Interactions have been reported between the phenolic compounds and the polysaccharides, the proteins and the lipids; hence, the phenolic compounds can be bound to all these molecule forms [55]. A range of technologies, including mechanical (e.g., milling, grinding, texture modification, and juicing), environmental (e.g., thermal including pasteurization and sterilization, freezing, drying, pressure, freeze-drying, pulsed electric field, and ultrasound), chemical (e.g., molecular bonding), and biotechnological (e.g., enzymatic treatments, fermentation, and encapsulations) are used in food processing to boost the availability and accessibility of polyphenols and other nutrients [56]. Here, we focus on the state-of-the-art opportunities of the most recent and advanced techniques. However, with a specific emphasis on chemical and biotechnological treatments that enhance the accessibility of polyphenols in food formulations. Below, we are therefore, briefly targeting thermal treatments, molecular bonding, the pulsed electric field, and ultrasound techniques before we discuss some major biotechnological methods such as the enzymatic treatments [55,57], fermentation [58], and encapsulation [59].

### 4.1. Non-Chemical Technologies

#### 4.1.1. Thermal Treatment

Thermal treatments of various types are among the oldest and most frequently used (cost-efficient) technologies applied by consumers at home (frying, boiling, steaming, oven cooking, microwave, and roasting) and in the food industry (sterilization, pasteurization, and buckling), which strongly impact the content and availability of nutrients and metabolites in various food items [60]. Temperature treatments of food are known to effect sensorial properties (flavour, taste, and texture), colour (e.g., through the Maillard reaction), shelf life by distorting pathogens, toxins, or inactivating enzymes, and nutrient absorption [14,18,25,60]. However, temperature treatments are also known to induce the formation of anti-nutritional or toxic compounds in some products and to influence the activity and potential of some compounds (e.g., Vitamin C and probiotic cells) in other outcomes [61]. In most of these cases, the advantages of thermal processing are considerably higher than the disadvantages [62]. Thermal treatment is used on a wide range of food items [60], although the impact and most suitable temperature vary due to various factors. Thus, a thermal treatment increased the polyphenol content in strawberry puree, but a similar increase was not observed if the puree was mixed with kale juice rich in proteins [63]. In fact, several studies have shown that the antioxidant activity of the polyphenols is diminished or inactivated by thermal treatment [63,64]. However, a designed thermal processing method has the potential to minimize such actions, although the food type, processing time, and involved processes, still have to be taken into consideration [14]. A designed thermal processing methodology (with 25 min boiling and 25 min steaming) was evaluated for cassava, with an increase in bio-accessibility of the phenolic content, due to alterations of the lignocellulosic structures, as a result [65]. Thermal processing was not found to effect the bio-accessibility of polyphenols in orange juice [66], in contrary to blackberry jam in which the bio-accessibility increased [14]. The differences in effects of thermal treatments on accessibility of polyphenols, may have a range of explanations, most of them relating to the bonds between polyphenols and other molecules in the food matrix, not least of which are fibers [67]. Table 1 summarizes some studies that investigate the effect of thermal treatment on polyphenol bioavailability.

#### 4.1.2. Pulsed Electric Field (PEF) and Ultrasonic Techniques

The PEF and ultrasonic techniques have been developed during recent years and are regarded as among the most promising novel technologies to extract and increase the accessibility of polyphenols in food formulations [73,74]. The use of the PEF technique results in electroporation of the cell walls and cell membrane, which means an irreversible permeabilization of these structures, by the placement of a food product between two electrodes [75]. Studies on beverages have shown diverging results as related to the accessibility of polyphenols while PEF treated; increased levels have been found in several cases [76,77,78,79] although some studies have indicated no changes [80,81]. PEF has been used as a pretreatment to increase bioactive compounds’ extraction yield from brewer-spent grain (BSG). In order to increase the amount of flavan-3-ols, flavonoids, phenolic acid derivates, and total free phenolic compounds, variables such as electric field strength E (0.5, 1.5, and 2.5 kV/cm), frequency (50, 100, and 150 Hz), and treatment duration (5, 10, and 15 s) were optimized [82]. The recovery of phenolic compounds from BSG and their subsequent purification for use as functional ingredients in the food and cosmeceutical industries is a crucial objective. The utilization of PEF as a pretreatment in BSG samples resulted in a 2.7-fold increase in total free phenolic compounds and a 1.7-fold increase in bound phenolic compounds under optimal conditions compared to extraction without PEF pretreatment. This improvement in phenolic recovery suggests the efficacy of PEF pretreatment in enhancing the extraction process [82]. Hiba N. Rajha and colleagues investigated the effects of PEF-assisted extraction compared with conventional extraction and extraction aided by infrared irradiation, ultrasound, and high-voltage electrical discharges (HVED) on the extraction of bioactive compounds from pomegranate peels. According to the study, both HVED and PEF treatments were highly effective for extracting phenolics from pomegranate peels. While HVED was more effective than PEF in terms of polyphenol recovery, PEF was found to be a less damaging and more selective pretreatment. Therefore, PEF is considered a more suitable option for industrial implementation [83]. The ultrasound treatment induces micro- to nanoscale structural changes to membranes and proteins through the generation of gas bubbles that grow and collapse [84]. The generation of molecular interactions through ultrasonic waves can lead to the disintegration of cell wall structures, reduction in particle size, and improved mass transfer across cell membranes. As a result, cracks may form due to the disintegration of cell walls, which can enhance tissue permeability and increase the release of targeted compounds [85]. In their study, Chao Chen and colleagues evaluated various factors to assess the effects of ultrasonic (US) pretreatment and temperature on the extraction kinetics of polyphenols from defatted oat bran. Specifically, they examined changes in phenolic profile, total phenolic contents, total avenanthramide contents, antioxidant activity, and β-glucan extraction rate using both conventional and US-assisted extraction methods [86]. In comparison to the traditional extraction approach, the results demonstrated that US pretreatment is more suitable for obtaining a higher yield of free phenolics with stronger antioxidant activities (ORAC) in a shorter period of time, whereas bound phenolic contents were reduced. Moreover, raising the extraction temperature greatly increased the extraction kinetics of free phenolics in defatted oat bran while decreasing the bound fractions. US pretreatment increased the initial extraction rate, but it had a temperature-dependent impact on the evolution and final values of free phenolic contents, antioxidant activities, and avenanthramides. Comparable outcomes were attained when anthocyanins from wine lees were extracted using both ultrasound-assisted extraction and natural deep eutectic solvents. The ideal conditions, such as an extraction period of 30.6 min and an ultrasonic power of 341.5 W, allowed for the highest amount of extracted compounds to be recovered [87]. As a result, the use of ultrasonic pretreatments shows great promise in lowering cell wall rigidity, which can improve the availability of essential compounds.

### 4.2. Chemical Technologies

#### Molecular Binding

The interactions in terms of chemical bond formation between polyphenols with a range of other molecules such as proteins, lipoproteins, minerals, dietary fibers, cellulose, etc., have been described in a wide range of studies, e.g., [6,88,89]. Despite the fact that this topic is well studied (some recent examples are shown in Table 2), and a variety of both experimental (e.g., spectroscopic methods including fluorescence emission, UV-vis adsorption, circular dichroism, Fourier transform infrared and mass spectrometry, nuclear magnetic resonance, X-ray diffraction, and light scattering techniques including small-angle X-ray scattering and small-angle neutron scattering, and calorimetric techniques) and computational methods (e.g., molecular docking, molecular dynamics, Monte Carlo simulations) have been utilized [89,90], mechanisms behind the formation and effects from various types of molecular bonds on bio-accessibility are not fully understood [6]. However, as bond formation between molecules, including the polyphenols, may have both positive and negative impacts on human health [6,91,92,93] increased knowledge and understanding may contribute to increased tailoring in future food matrices of such interactions.

### 4.3. Biotechnological Technologies

#### 4.3.1. Enzymatic Treatments

Enzymatic hydrolysis, using substrate-specific enzymes, has been shown as a particularly effective method to reduce matrix interactions hindering the release of polyphenols [55]. Although, limited in vivo studies are available describing bio-accessibility and uptake of polyphenols in the human intestine after enzymatic treatment [56], a range of studies have indicated the accessibility of the polyphenols after enzymatic treatment by their availability [55]. The methods of enzymatic hydrolysis used for this purpose include mainly the use of carbohydrases such as cellulases, hemicellulases and pectinases, which disrupt the plant cell wall, thereby decreasing the binding affinity for the polyphenols [30,102].

Grape pomace produced during grape juicing and winemaking contains a relevant amount of underutilized phenolic compounds, which are polymeric and have lower bioefficacy than their monomeric and aglycone derivatives. A cellulase treatment on the grape pomace is shown to release p-coumaric acid and malvidin-3-*O*-glucoside, and optimally, this treatment may be combined with a tannase treatment to release gallic acid [103]. The bioavailability of phenolic compounds from wheat, rice, and oat bran has been shown to increase by combining optimized enzymatic treatment with suitable processing conditions. Thus, the bio-accessibility of ferulic acid in wheat bran has been increased using a 3-step procedure comprising autoclaving (20 min/120 °C), enzymatic pretreatment with protease and a-amylase, and finally, further enzymatic treatment with driselase (cell wall degrading enzyme pool containing cellulase, hemicellulase, pectinase, and others), xylanase, or feruloyl esterase (FAE) [104]. In this study, FAE was the most efficient enzyme to obtain ferulic acid (12.8-fold), and its effectiveness was significantly increased when combined with the enzymatic pretreatment. In rice bran, the amount of total phenolics was 50% higher after processing through a gelatinization process (10 min/100 °C), liquefaction at 70 °C using α -amylase, and a further enzymatic step. During the enzymatic treatment of rice bran with glucoamylase, proteases, and cellulase, incubation parameters were set as 57.5 °C for 190 min [102]. To extract polyphenols from oat, a combined method of cellulase and protein precipitation has been used, and this treatment increased the phenolic content by 14% [105]. Other carbohydrases such as β-glucanase, α-amylase, and amyloglucosidase have also been reported to have beneficial effects influencing antioxidant and anti-microbial activity [106].

A diet rich in phenolic compounds does not necessarily mean a healthy diet, as the chemical composition and structure of the phenolic compounds play a key role in metabolism and hydrophilic polyphenols cannot penetrate the gut by passive diffusion [4]. In this context, tannases are widely employed in a food context as they reduce the tannin content and catalyze hydrolysis reactions. Studies have shown that grape pomace treated with tannase resulted in an increased content of the aglycone quercetin, followed by an increase in gallic acid [58]. Similarly, tea extracts treated with tannase boosted polyphenolic composition with increased content of gallic acid, caffeic acid, and flavonol aglycones [107]. These studies suggest that tannase improves both bio-accessibility and bioavailability of phenolic compounds.

In general, the bio-accessibility of the phenolics is best improved by combining already established food processing unit operations and enzymatic treatments targeting the release of these compounds by carbohydrases and other enzymes. An optimized process where enzymes are used contributes to reducing treatment steps and does not include buffers, strong acids, or alkali. Thus, the application of hydrolytic enzymes contributes to the improved yield and health benefits of phenolic compounds in scalable processes.

#### 4.3.2. Fermentation

Enzymes secreted by bacteria, fungi, or yeasts are known to degrade cell walls, thereby releasing phenolic compounds, which results in an increase in availability of these compounds [54]. Fermentation utilized in food processing traditionally builds on the activities from microorganisms, and thus, such treatment also releases the phenolic compounds from the food matrixes. After fermentation, the levels of extractable phytochemicals with high bioactivity are expected to have increased [108], and fermentation using lactic acid was shown to increase the antioxidant activity of the polyphenols [23].

However, the effect of the fermentation process has been found to be influenced by several factors, such as time, methods, matrix, and even the polyphenols themselves. During the fermentation process, natural enzymes found in the raw material may come in contact with bioactive compounds, thereby reducing the availability of the polyphenols, and flavours might be developed [109,110]. Polyphenols of the raw material have been shown to affect the production of lactic acid and interfere with the fermentation, e.g., polyphenols from grapes were shown to inhibit the production of lactic acid in wine production [111]. Usually, milk and dairy products present an ideal matrix for fermentation due to the amount of bioactive compounds present; although the lactic acid influences the texture, flavour, and nutritional value of the end-product [24]. The metabolic activity of the starter culture used for the fermentation process, is closely related to the success of the fermentation and to the release of phenolic compounds [112]. Besides releasing phenolic compounds, fermentation is beneficial because it produces anti-microbial compounds that help eliminate food-borne pathogens and contaminants that might degrade the product. Lactic acid, for example, can enter bacteria and kill the microorganism at decreased pH [38].

Effects on polyphenol content from various fermentation methods are summarized in Table 3. As can be concluded from Table 3, optimization of the fermentation process is key for a high level of bioactive compounds in the final product [113,114].

Despite the fact that a high number of studies have indicated an impact on phenolic compound content and availability by the use of fermentation, only a few in vivo studies have evaluated the bioavailability and metabolic uptake of these compounds after fermentation [56]. Until now, most in vivo studies have focused on isoflavones in soy [56], and an improved bioavailability and bioactivity have been reported in products after fermentation [124,129,130]. Most of the studies investigated how fermentation affected the isoflavones in soy products’ absorption and metabolism. Since they are more lipid-soluble and hence more easily able to pass through the intestinal barrier than the original isoflavone glucosides, isoflavone aglycones found in fermented food showed enhanced bioavailability and bioactivity [131]. Another human investigation found that eating fermented soybeans led to alterations in soybean isoflavones that increased simple and acylated glucoside levels, which led to the same result, i.e., faster absorption and higher bioavailability of certain metabolites in plasma [132].

#### 4.3.3. Encapsulation

Despite the known excellent biological activity of the polyphenols, and the increased interest to use them as plant-based natural food additives, e.g., for preservation, uses are often hampered by their instability in food formulations and in functional foods [33]. Temperature, oxygen, and light have been found to induce degradation of the polyphenols during storage and processing [133]. Furthermore, bioactivity is decreased in vivo in the gastrointestinal tract by enzymes or low pH [134]. Various methodologies have been evaluated in order to maintain stability, bioactivity, and bioavailability of active ingredients, of which encapsulation technologies at the micro- and nanoscales have been reported highly efficient [135,136]. Encapsulation of the polyphenols has a beneficial influence on their shelf life and bioaccessibility. Different matrices have been utilized to protect the polyphenols from digestion, and these matrices also have the benefits of contributing to controlled release properties, which might lead to improved bioavailability [136]. Over the years, a range of encapsulation methods have been reported and used [137,138]. These techniques include spray drying, freeze drying, electrospinning, electrospraying, fluidized bed coating, and liposome entrapment and emulsion methods [137]. Similarly, a variety of biobased materials have been used for the encapsulation of polyphenols, of which some are already extensively applied in the pharmaceutical field to enhance the absorption of bioactive compounds [139]. Biobased materials that have been used for encapsulation of polyphenols include chitosan, alginate, a combination of maltodextrin and gum arabic, starch, and oxidized gellan gum and resistant starch composites, as described below.

The natural polycationic biopolymer chitosan has been utilized as a nano- or microcarrier due to its biodegradability and biocompatibility [15]. Chitosan is positively charged and contains protonated amino groups that interact with negatively charged groups of polyphenols [140]. These properties contribute to a high degree of encapsulation which enhances the bioavailability and a sustained release of the active ingredient [140]. Application of chitosan nanocarriers was reported to enhance the absorption and bioavailability of tea polyphenols, especially in the gastrointestinal tract [141].

Chitosan has been found to be a suitable matrix material for the microencapsulation of curcumin, which improves the therapeutic effects on many chronic diseases, including bacterial infection, wound healing, and Alzheimer’s disease [142]. Thus, the bioavailability of cucumin encapsulated in chitosan nanocarriers was found to increase nine-fold as compared with non-encapsulated curcumin in in vivo mice experiments [143].

Alginate is a linear 1,4-linked copolymer of α-L guluronic and β-D mannuronic acids, that cross-links non-covalently with cations like calcium chloride and polycations like chitosan, which results in gelation. Microencapsulation of extruded phenolic extracts from *Clitoria ternatea* petal flower, utilizing alginate and calcium chloride as a matrix material, resulted in an improved antioxidant capacity, pancreatic α-amylase inhibitory activity, and bile acid binding capacity of the phenols after gastrointestinal digestion [144]. Furthermore, a stable antioxidant capacity was the result of the encapsulation of lemon balm utilizing calcium alginate particles [145]. Alginate and calcium chloride were used to microencapsulate thyme essential oil emulsion microspheres by ionic gelation, which contributed as a significant antimicrobial effect to Gram-positive bacteria [146]. Swelling and degradation of the matrix were found to be important parameters for the release and delivery of the thyme oil when alginate-soy protein isolate beads where used for its encapsulation [147]. Furthermore, encapsulation of curcumin sodium alginate/zinc oxide composite hydrogel beads contributed protection to light degradation of the curcumin, which resulted in a high antioxidant capacity. The release profile of the encapsulated curcumin demonstrated a good pH sensitivity and controlled-release capacity [148].

The combination of maltodextrin and gum arabic for encapsulation has had more limited investigation than the above-described chitosan and alginate. However, the most suitable composition of maltodextrin and gum arabic to encapsulate grape pomace, as a residual product from the wine industry, has been evaluated [149].

Beads of pea and mung bean starch were found to have a high loading efficiency and quick release capacity in the intestine of antioxidant active polyphenols, thereby indicating the suitability of starch as a carrier for active ingredients [150].

Ionic cross-linking of oxidized gellan gum and resistant starch was used to fabricate composite hydrogel beads for resveratrol encapsulation, and demonstrated good stability and sustained release in simulated gastric and intestinal fluids, respectively [151].

Thus, the field of encapsulation of polyphenols to improve the stability and bioavailability of these bioactive compounds is growing rapidly; although the majority of the studies have focused on the release of compounds that have therapeutic functions in the human intestine. Encapsulation to increase the accessibility of polyphenols from food to the human metabolism has been less studied. As for all novel products, the encapsulation of natural polyphenols necessarily need to be both efficient and cost effective to be competitive to other products [152].

## 5. Future Opportunities to Increase Accessibility of Polyphenols from Food

Based on the fast-emerging knowledge in the field of polyphenols and their effects on food characteristics and human health, and the large variety of novel technologies developing in food science, the opportunities to increase the accessibility of polyphenols from food products has never been greater. However, both the food products and the human intestine system are complex environments that influence the accessibility of the polyphenols, as described above. Recent studies have indicated the presence of phenolic compounds in all fractions after protein fractionation, indicating their bindings to other molecules even under relatively harsh processing conditions [153]. In the human body, the effect of digestive enzymes and pH conditions account for bio-accessibility and bioavailability of phenolic compounds as well as interactions with other molecules [154].

**Figure 2:**
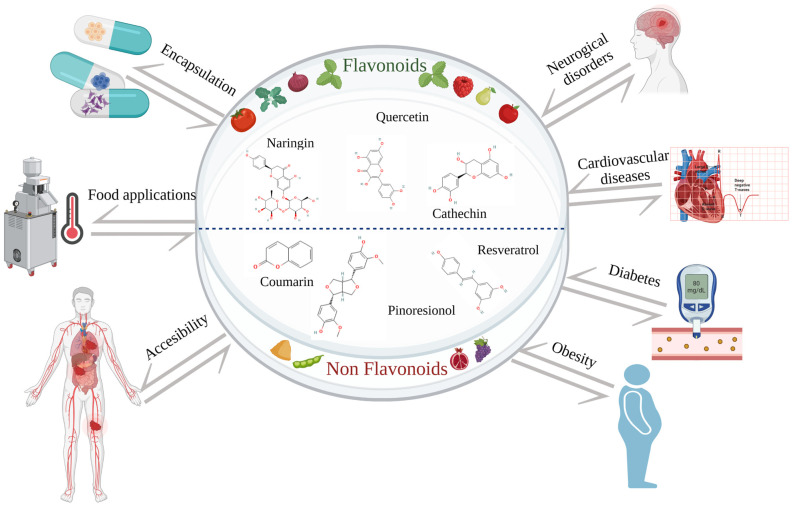
Future directions to increase accessibility of polyphenols from food, and their impact on human health.

Encapsulation of polyphenols and other molecules has been shown, not least by medical science, as a form of delivery of the molecules in the right shape and to the right place in the human body. Encapsulated phenolic compounds in food products have been evaluated in only a few studies [137]. The development of bioactive compound-enriched food through the use of encapsulation technology is an interesting future track for the food industry to enter. This track has the opportunity to contribute novel food products with high nutritional value and quality, as the safety and cost aspects are simultaneously taken into consideration [137]. Despite this facts, it is important to keep in mind the importance of the food matrix design [57]. Furthermore, encapsulated phenolic compounds (Figure 2) need to be stable both during food processing and food matrix conditions [137]. The food matrix design is also of relevance to understand opportunities to supply nutritious and polyphenol rich food products to the consumer when more traditional processing methods are used than the encapsulation of molecules. For an improved understanding of interactions between molecules in the food matrix and along the interior human body after consumption, simulation methods in combination with experimental methodologies are advised [155].

## 6. Summary and Conclusions

Polyphenols are extremely important compounds in the human diet that contribute to the promotion of human health and to the improved quality of human food items. Polyphenols have an immunomodulatory role, and their intake correlates with a decrease in incidences of chronic illnesses such as cardiovascular diseases, diabetes mellitus, and cancer, while they simultaneously protect food products by their anti-microbial and antifungal properties. The bio-accessibility and bioavailability of polyphenols are key to the impact of their uses, and these characteristics are highly connected to the structures of the polyphenols but also to their binding to other molecules in the matrix. The food matrix should therefore be designed to allow bio-accessibility of polyphenols. To design the food matrix, a combination of traditional and novel methodology, e.g., thermal, chemical, and biotechnological, may be utilized. Among the novel methodologies, encapsulation, currently mainly used for delivery of compounds in human medical science, is an emerging and interesting technology also to be used within the food industry. However, although the polyphenols can be encapsulated for suitable delivery within the human bowl or intestine, they still need to be properly incorporated into the food matrix, so that health promoting, tasty, safe, and cost-effective food items are created. Thus, enzymatic treatment or fermentation might be used to release the polyphenols before they are encapsulated, and then thermal and chemical processes might be suitable for the incorporation of the encapsulated molecules in the food matrix. Thus, in the future highly nutritious polyphenol bio-accessible food products will need to be more precisely tailored compared to current food products.

## Figures and Tables

**Table 1:** Effect of thermal treatment on polyphenol bioavailability.

Food	Processing Conditions	Effect of Polyphenols Bioavailability	References
Cassava	Boiling (~98 °C, 25 min) and steaming (over boiling water, (~100 °C, 25 min)	↑ total phenolic bio-accessibility total polyphenols bio-accessibility >70% ↓ total phenolic content after the in vitro digestion, 12.10 mg GA/100 g (boiling) and 19.24 mg GA/100 g (steaming) before the in vitro digestion16.59 mg GA/100 g (boiling), 25.81 mg GA/100 g (steaming)	[65]
Baobab (*Adansonia digitata*) juice	Pasteurization 72 °C, 15 s	↓ total phenol content significantly ↓ procyanidin B2 by 12.6% ↑ (-)-epicatechin by 10.9%	[64]
Orange juice	Thermal treatment (85 °C, 1–15 min; 99 °C, 10 s)	protect total polyphenol bio-accessibility	[66]
Black mulberry	Jam processing (95 °C, 25 min)	↑ total polyphenol bio-accessibility ↓ flavonoids bio-accessibility ↓ total phenolics (by 88%), total flavonoids (by 89%), anthocyanins (97%), and antioxidant capacity (88–93%) ↑ recovery of bioaccessible total phenolics, ↑ anthocyanins and total antioxidant capacity (having the recovery values of 16%, 12%, and 37% for TPC, TMA, and TAC (ABTS) ↓ of bioaccessible total anthocyanins (5%) ↑ the recovery of bioaccessible total antioxidant capacity: CUPRAC (36%)	[68]
Mulberry juice-enriched dried minced pork slices	dried 10 h at 40 °C followed by baking 3 min at 150 °C	↑ phenols retention rates (54.84% polyphenols, 39.1% flavonoids, and 59.62% anthocyanins)	[69]
Apple pomace	T = 30, 50, 80, 80, 100 and 100 °C (from feed to die) Feed rate = 30 kg/h screw speed = 370 rpm	significantly ↑ the antioxidant activity (ORAC) in the in vitro gastrointestinal digestion total extractable polyphenols, measured as gallic acid equivalents, ↓ by extrusion (barrel moisture 30%) but was not affected by extrusion at lower barrel moistures (15% or 20%)	[70]
Juçara (*Euterpe edulis* Martius)-based smoothie (juçara (20%), banana (40%) and strawberry (40%)	Pasteurization 90 °C, 35 s	↑ total phenolic compounds bio-accessibility (47%), ferulic (16%), and ellagic (80%) acids in vitro intestinal bio-accessibility varied from 20 to 47% in vitro gastric and intestinal digestion bio-accessibility of the phenolic compounds was ↑ in the intestinal digest, due to the increase of pH ↑ more in the pasteurized smoothies than in the control sample	[71]
Strawberry-kale-mix (strawberry) puree 20% and kale juice 80%)	Thermal treatment (70 °C, 2 min)	↓ free anthocyanin content after gastric digestion with 44% for PR and 48% for CG minor ↑ (8% ± 2%) for CG and 16% ± 2% for PR and ↓ by 15% to 18% in PG, PMG and PAG	[63]
Wheat bran (WB) and oat bran (OB)	Thermal treatment 10 min at 80 °C	↑ total phenolic content for WB (ferulic acid + 39.18%, vanillic acid + 95.68%, apigenin–glucoside + 71.96%, p-coumaric acid + 71.91%) and of OB (avenanthramide 2c + 52.17%, dihydroxybenzoic acids + 38.55%)	[72]

GA—gallic acid; ↑—increase; ↓—decrease; PR—pelargonidin-3-*O*-rutinoside; CG—cyanidin-3-*O*-glucoside; PMG—pelargonidin-3-*O*-malonylglucoside; PAG—pelargonidin-3-*O*-acteylglucoside.

**Table 2:** Polyphenols bound with different compounds and their availability.

Polyphenols	Bonding Compounds	Interactions/Process	Test/Effect/Availability	References
Polyphenols from black carrots (anthocyanins and phenolic acids)	Dietary lipids from coconut oil, sunflower oil, and beef tallow	Hydrophobic interactions and hydrogen bonds	simulated in vitro gastrointestinal digestion and colonic fermentation ↑ accessibility	[94]
Curcumin	Lipids (milk fat)	Hydrogen bond interactions	after in vitro gastrointestinal digestion, 11% of the curcuminoids delivered in yoghurt was degraded compared to <1% for curcuminoids in aqueous dispersion, but was 15-fold more bio-accessible than curcuminoids in aqueous dispersion	[95]
Rosmarinic acid	Whey protein (α-lactalbumin, β-lactoglobulin, and Lactoferrin)	Hydrogen and hydrophobic bonds and van der Waals interaction	↓ rosmarinic acid activity in the presence of milk proteins ↑ in protein stability	[43,96]
Oat polyphenols	Casein and whey protein	Covalent interaction Hydrogen bonds	in vitro gastric and pancreatic digestion ↑ antioxidant activity and bio-accessibility of oat phenolics when mixed with milk whey protein	
Tea polyphenols	β-Lactoglobulin caseino-macro-peptide	Hydrophobic interactions and hydrogen bonds	maintain anti-proliferative activity against different tumour cell lines ↑ accessibility, synergetic effects	[97]
Green tea epigallocatechin- 3-gallate (EGCG)	Bovine α-lactalbumin (ALA)	Hydrophobic interactions	non-covalent interactions, binding affinity, and binding site between ALA and EGCG ↑ biological activity of EGCG	[98]
Rutin	Bovine β-lactoglobulin (BLG)	Hydrogen and hydrophobic interaction	BLG can serve as a suitable transporter for the hydrophobic ligand	[99]
Resveratrol	Gliadin	Colloidal complexes	in vitro gastrointestinal digestion model bio-accessibility ↑ lipid oxidation stability	[100]
Grape polyphenols	Cellulose–lignin hydrogels	Hydrogen bond interactions	depending on the lignin content, hydrogels can control the release of polyphenols	[101]

↓—decrease; ↑—increase; ALA—α-lactalbumin; EGCG—epigallocatechin-3-gallate; BLG—β-lactoglobulin.

**Table 3:** Impact of different fermentation processes on bioavailability of polyphenols.

Fermentation Type	Matrix	Microorganisms	Influence on Polyphenols	Determination Method	References
Lactic	Orange juice milk-based beverage	*L. brevis* POM, *L. plantarum* TR-71, TR-14	↑ total polyphenol content	Spectrometry	[115]
Intestinal fermentation	Water-insoluble cocoa fraction	*Bacteroides–Prevotella* spp., *Bifidobacterium* genus, *Lactobacillus–* Enterococcus group, *Clostridium histolyticum* group	↑ polyphenol content	LC/MS/MS	[116,117]
Solid-state fermentation	Sul 1 cacao	*Ceratobasidium theobromae*	↓ polyphenol content and methylxanthines (theobromine and caffeine)	NP-HPLC RP-HPLC	[109]
Solid-state fermentation	Dandelion	*L. plantarum* (CGMCC No. 1.12934) *S. cerevisiae* (CGMCC No. 2.1190)	↑ polyphenol content	UPLC-ESI-MS/MS	[113]
Lactic acid fermentation	Kiwifruit	*L. plantarum*	↑ total phenolic content	Spectrometry	[23]
Solid-state fermentation	Ginger	*S. cerevisiae*, *Bacillus licheniformis*, *B. pumilus*, *B. safensis*	↑ polyphenol content	Titration	[54]
Solid-state fungal fermentation	Green coffee beans	*Aspergillus luchuensis* Inui (JCM 22239), *A. Oryzae* (Ahlburg) Cohn var. Brunnues Murakami (JCM 2059), *Mucor plumbeus* Bonorden (JCM 3900)	↓ polyphenol content	HPLC	[118]
Solid-state fermentation	Lentil cultivars	*Aspergillus awamori* (MTCC 548)	↑ polyphenol content	HPLC	[119]
Lactic acid fermentation	Avocado fruits	*L. plantarum AVEF17*	↑ polyphenol content	Spectrometry	[120]
Malolactic fermentation	Sea buckthorn, Sea buckthorn-apple juice	*L. plantarum*, *Argentoratensis*, *Oenococcus oeni*	↑ polyphenol content	UPLC-PDA UPLC	[121]
Submerged fermentation	Wheat straw	*Inonotus obliquus*	↑ polyphenol content	HPLC-DAD ESI–MS/MS	[122]
Fungal fermentation	Turmeric	*Monascus purpureus*, *Eurotium cristatum*	↑ polyphenol content	LC-QTOF-MS/MS	[123]
Solid-state fermentation	Mixed grains	*Bacillus amyloliquefaciens* 245	↑ polyphenol content	Spectrometry CE-TOF-MS	[124]
Solid-state lactic acid fermentation	Wheat bran	*L. rhamnosus*	↓ total phenolic content slightly ↑ polyphenol content	Spectrometry	[125]
Controlled alcoholic fermentation	Orange juice	Saccharomycetaceae *Pichia kluyveri*	↑ polyphenol content	UHPLC	[108]
Co-culture submerged fermentation	Extruded brown rice	*L. plantarum*, *L. fermentum*, *Saccharomyces cerevisiae*	↑ total polyphenol content	Spectrometry	[126]
Solid-state fermentation	Whole soybean flour	*L. casei*	↑ total polyphenol content	HPLC	[127]
Solid-state fermentation	Seaweed	*Aspergillus oryzae*	↑ polyphenol content	LC-MS/MS	[128]

↑—increase; ↓—decrease; LC/MS/MS—liquid chromatography tandem mass spectrometry; NP-HPLC—normal phase high-performance liquid chromatography; RP-HPLC—reversed phase high-performance liquid chromatography; UPLC-ESI-MS/MS—ultra-performance liquid chromatography–electrospray ionization–tandem mass spectrometry; HPLC—high-performance liquid chromatography; UPLC-PDA—ultra-performance liquid chromatography coupled with photodiode array; UPLC—ultra performance liquid chromatography; HPLC-DAD—high-performance liquid chromatography with photodiode-array detection; ESI–MS/MS—direct insertion electrospray ionization mass spectrometry; LC-QTOF-MS/MS—qualitative tandem liquid chromatography quadrupole time of flight mass spectrometry; CE-TOF-MS—capillary electrophoresis time of flight mass spectrometry; UHPLC—ultra-high-performance liquid chromatography.
